# 骨髓造血衰竭伴躯体畸形

**DOI:** 10.3760/cma.j.issn.0253-2727.2022.03.012

**Published:** 2022-03

**Authors:** 向荣 胡, 莉 张, 贝贝 赵, 园 李, 小霞 李, 凤奎 张, 馨 赵

**Affiliations:** 中国医学科学院血液病医院（中国医学科学院血液学研究所），实验血液学国家重点实验室，国家血液系统疾病临床医学研究中心，天津 300020 Anemia Therapeutic Center, State Key Laboratory of Experimental Hematology, National Clinical Research Center for Blood Diseases, Institute of Hematology & Blood Diseases Hospital, Chinese Academy of Medical Sciences & Peking Union Medical College, Tianjin 300020, China

患者，男，17岁。因“间断皮肤瘀斑5年，外伤出血难止1个月”于2020年9月就诊于我院。5年前患者无明确诱因反复出现下肢皮肤瘀斑及皮下血肿，血常规：WBC 6.3×10^9^/L，HGB 116 g/L，PLT 80×10^9^/L。骨髓涂片示有核细胞增生明显活跃，粒系占0.380，红系占0.260，巨核细胞120个，以颗粒型巨核细胞为主，血小板少见。疑诊原发免疫性血小板减少症（ITP），未行特殊治疗。2020年8月前患者出现皮肤瘀斑及手指割伤后出血难止，血常规示WBC 5.66×10^9^/L，HGB 106 g/L，PLT 37×10^9^/L，凝血功能无明显异常，输注血浆后渐止血。为进一步诊治入我院。既往史：患者为早产儿，自幼生长发育较同龄人迟缓。有隐睾病史，4岁时行双侧隐睾下降固定术；8岁时诊断眼球震颤；频发呼吸道感染，9岁时行双侧扁桃体切除术，术中及术后无明显出血。16岁诊断少精症。头孢类抗生素过敏。家族史：父母为非近亲结婚，父亲身材矮小，身高155 cm，存在眼睑下垂、耳位低、口齿欠清。母亲身高158 cm，外表无明显异常。否认血细胞减少及出血性疾病家族史。

入院查体：身高158 cm，体重50 kg，神志清楚，对答正常。营养中等，轻度贫血貌，下肢皮肤散在瘀斑及皮下血肿，以膝关节附近为主，无关节畸形；血肿以右侧股骨下缘前侧为重。周身浅表淋巴结未触及肿大。高额头；内眦皱褶，上睑下垂；鼻梁凹陷；高腭，牙齿错位咬合；伸舌居中，舌系带短致舌尖前伸受限，不能超越上下唇缘，口齿不清晰；下颌小，耳位低，蹼颈，后发际低。漏斗胸，乳距增大。胸骨无压痛，双肺呼吸音清，心律齐，未闻及病理性杂音。腹软，肝脾不大。脊柱侧弯。双手手指过伸，双足趾细长。父亲存在类似外观异常。

入院血常规：WBC 4.75×10^9^/L，中性粒细胞绝对计数（ANC）2.73×10^9^/L，单核细胞比例4.6％，单核细胞绝对计数0.22×10^9^/L，HGB 114 g/L，PLT 35×10^9^/L，网织红细胞计数（Ret）120.4×10^9^/L。肝肾心功能、叶酸、维生素B_12_、铁蛋白及血清铁四项未见异常，EPO 76.16 IU/ml。凝血指标：纤维蛋白原2.02 g/L，PT 12.6 s，APTT 30.7 s。免疫相关检查、阵发性睡眠性血红蛋白尿（PNH）克隆检测、直接抗人球蛋白试验、感染相关标志物、血小板特异性抗体检测均未见异常。静脉血彗星试验及丝裂霉素C（MMC）试验结果回报未见明显异常。尿、便常规无明显异常。骨髓象：粒（+），油（+），增生活跃，粒系占0.410，红系占0.260，全片巨核细胞46个（图1A）。骨髓活检：增生较低下，细胞面积30％～40％，粒红比例减小，巨核细胞少见，网状纤维染色（0级）（图1B）。免疫分型未见异常。小巨核细胞免疫酶标（CD41）：全片巨核细胞27个，正常巨核细胞19个，双核细胞免疫巨核细胞5个，多核巨核细胞2个，大单圆核小巨核细胞1个。染色体核型：46,XY[20]。超声：肝实质回声增强；胆胰脾未见明显异常；左肾多发囊肿，右肾未见明显异常；三尖瓣少量反流。心电图未见异常。

**图1:**
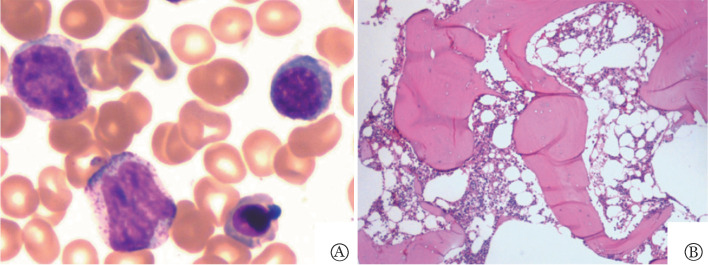
患者骨髓增生情况 A：骨髓涂片（瑞氏-吉姆萨染色，10×100）示增生活跃，三系增生、血小板减少骨髓象；B：骨髓活检（HE染色，10×10）示增生较低下（30％～40％），粒红比例减小，巨核细胞少见

## 第一次临床讨论

患者青少年男性，自幼发病，以出血为主要表现，伴有躯体发育异常。血常规示轻度贫血、血小板减少、网织红细胞轻度升高，骨髓活检增生低下、巨核细胞少见，需与以下疾病鉴别。

1. ITP伴失血：患者以血小板减低起病，近期出血后发现轻度贫血伴网织红细胞增高，脾脏无明显肿大，需排除ITP合并失血可能。患者骨髓穿刺未见巨核细胞产板不良，活检示巨核细胞减少，免疫相关检查及血小板特异性抗体检测均无异常，不支持ITP；同时，患者自手指较多量出血至来我院就诊，间隔已1个月余，期间未再发生严重出血，而患者HGB始终未能恢复正常，骨髓红系比例无明显升高，提示骨髓对贫血代偿不全；故而排除ITP伴失血。

2. Evans综合征：患者轻度贫血伴有网织红细胞增高，血小板减少，应除外Evans综合征。该患者自发病以来无黄疸表现，病前无感染、用药、自身免疫异常及其他可能引起溶血或血小板减少的证据，入院后查抗自身红细胞抗体（直接抗人球蛋白试验）和血小板抗体均为阴性，胆红素、LDH均在正常范围，而且骨髓红系无代偿性增高，巨核细胞减少。综合分析，无溶血及ITP的证据，除外Evans综合征可能。

3. 骨髓造血衰竭性疾病：患者血细胞轻中度减少，年龄校正后的骨髓增生程度减低，巨核细胞减少，未发现其他引起血细胞减少的疾病，提示患者存在骨髓造血衰竭（BMF）。造血衰竭性疾病分为先天性和获得性。先天性骨髓造血衰竭（IBMF）由基因突变导致，可有家族史，部分患者合并躯体发育畸形。获得性再生障碍性贫血为免疫异常引起造血干祖细胞损伤，患者一般无发育异常和类似疾病家族史。该患者少年时期发病且伴有躯体畸形，其父亲亦存在类似外观异常，首先考虑IBMF可能。IBMF是一组异质性遗传疾病，包括Fanconi贫血（FA）、先天性角化不良（DC）、Shwachman-Diamond综合征（SDS）等。FA在IBMF中相对多见，患者常表现为躯体异常伴轻中度的血细胞减少，其特征是染色体不稳定性及DNA修复缺陷，彗星试验及MMC试验检测染色体断裂可初步筛查；DC是一种罕见的遗传性端粒病，特征表现为皮肤黏膜三联征：角化不良、黏膜白斑和皮肤色素沉着；SDS是常染色体隐性遗传性疾病，表现为胰腺外分泌功能障碍、骨骼畸形和BMF。本例患者临床上无皮肤黏膜异常、角化不良及胰腺外分泌障碍等DC和SDS疾病证据。虽未发现染色体断裂试验异常，但该试验的敏感性仅60％，需进一步行基因检测以除外IBMF。

4. 艾-唐综合征：艾-唐综合征是遗传性结缔组织病，其特征是胶原蛋白合成异常，表现为皮肤及关节的高延展性、组织易碎性及伤口愈合较差。该患者以皮肤瘀斑及出血难止为主要表现，双手手指过伸、双足趾细长、脊柱侧弯，需除外艾-唐综合征。然而该患者未见疼痛、皮肤高延展性及疤痕形成不良，也没有相关家族史。可进一步完善基因突变筛查以排除。

5. 家族性血小板减少症：家族性血小板减少症是罕见的遗传性血小板异常疾病，表现为血小板数量减少，并往往伴有血小板功能异常。患者常表现为自幼有不同程度的出血倾向，并有血小板减少的家族史。该少年患者以血小板减少起病，应鉴别其他疾病如家族性血小板减少症，然而患者父母血常规检查均未发现异常，未找到血细胞减少的家族史证据，考虑该病可能性不大，可通过基因检测排除。

## 第二次临床讨论

患者外周血二代基因测序检查结果回报，未检测到IBMF、艾-唐综合征和家族性血小板减少症相关基因突变，而检出杂合性PTPN11基因错义突变[c.184T>G（p.Y62D）]，为文献报道的努南综合征（NS）的热点突变。对其父母行基因测序验证，证实突变来源于父亲，母亲为野生型（图2）。复习患者疾病表型，其躯体发育异常与NS表型高度相符，有基因热点突变证据，且突变来源的患者父亲亦存在类似表型，因此患者确诊为NS。

**图2:**
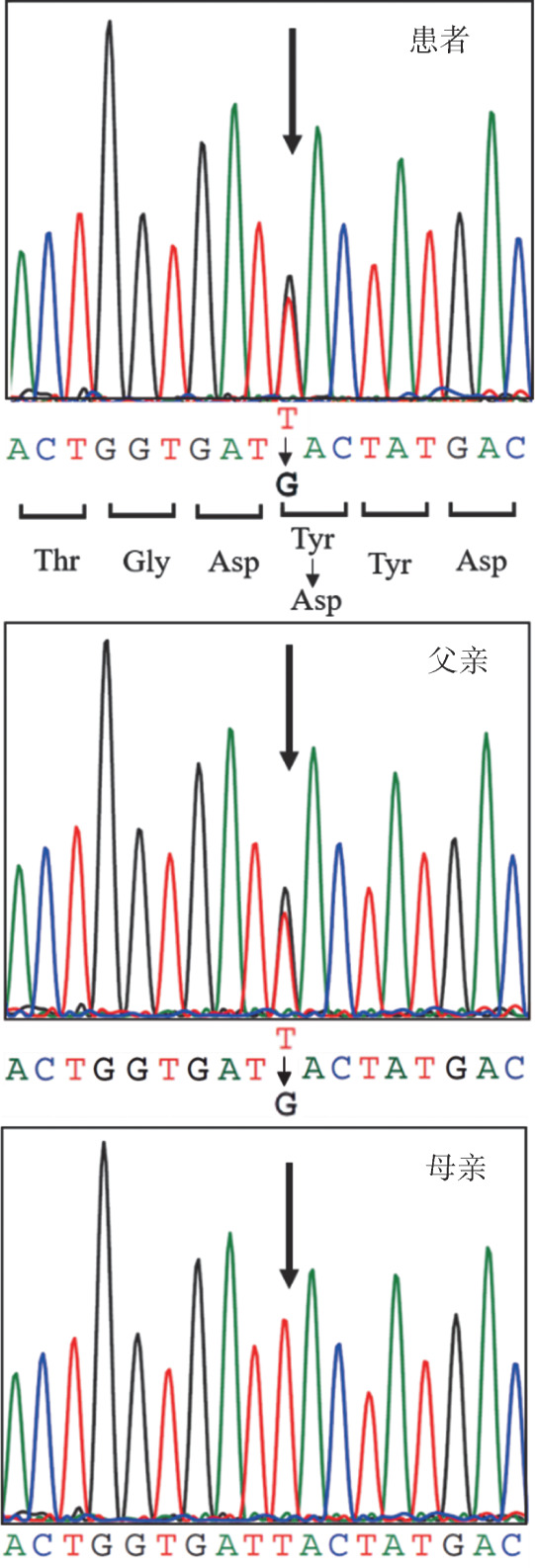
基因测序结果显示患者和其父亲有相同位点的杂合PTPN11基因错义突变（c.184T>G, p.Y62D, 箭头所示为突变位点）

NS是一种常染色体显性遗传病，可为家族性聚集或散发。大多数与NS相关的基因都参与编码RAS-MAPK信号传导通路中的关键蛋白，如PTPN11、SOS、KRAS、NRAS等。NS临床表现及疾病严重程度异质性大，病变常涉及多个系统。身材矮小最为常见，常存在面部和骨骼等畸形，如蹼颈、耳朵低位并向后旋转、上睑下垂、乳距增大、鸡胸或漏斗胸、脊柱畸形、肘外翻、膝外翻等。95％的NS患者存在至少一种眼部异常表现，80％的男性患者存在单侧或者双侧的隐睾症，少数患者存在肾脏异常。NS是除唐氏综合征外最常引起先天性心脏病的综合征。智力低下的NS患者约占20％，一部分患者还会出现社交认知障碍、情绪识别障碍、语言障碍等情况。此外，NS患者罹患肿瘤的风险较常人增加3～5倍。NS患者往往因为生长发育过程中的异常而在儿童期被识别，结合基因检测而确诊。本例患者有隐睾病史，同时存在身材矮小、发育迟缓、面部和骨骼畸形、眼球震颤等临床表现，但既往多次就医，均未能诊断，在我院亦通过基因检测方得以确诊，提示临床医师对于该病认识不足，未能及时识别。

NS无特异性治疗手段，多为对症和支持治疗。儿童期应注意听力测试、眼科检查和心脏评估。对于隐睾症男性，如睾丸未降，应在一岁左右行睾丸固定术，减少成年后睾丸癌风险。生长激素的治疗目前仍存在争议，有文献报道重组人生长激素（rhGH）治疗可显著提升NS患儿的身高，并且青春期前越早治疗且疗程越长越可以实现身高发育的最大化。部分PTPN11突变患者存在生长激素不敏感，治疗效果不佳，这部分患者常有正常或者轻度增高的生长激素水平和低浓度的血清胰岛素样生长因子1（IGF-1），对于此类患者可采用替代的重组人IGF-1治疗。本例患者为青少年男性，身材矮小，建议其至儿科和内分泌代谢科进一步咨询生长激素治疗。

## 第三次临床讨论

针对患者的血液学表现和躯体发育畸形，是否可考虑一元论解释？

复习文献得知，20％～65％的NS患者合并血液系统异常，其中出血异常最为常见，1/3的患者有凝血功能障碍，如凝血因子Ⅷ、Ⅺ、Ⅻ的缺乏和血小板功能的异常等。因患者反复出现皮下血肿及皮肤大片瘀斑，APTT、PT检测未见异常，我们进一步查血管性假性血友病因子抗原、血管性血友病因子活性测定、�因子筛选及血栓弹力图，以明确是否合并有出凝血异常性疾病。结果回报均无异常，未找到出凝血异常的证据。NS患者罹患血液系统肿瘤如幼年型粒-单核细胞白血病（JMML）、急性髓系白血病（AML）、急性B淋巴细胞白血病等风险增加，但未见合并造血衰竭的报道。该患者二代测序未检出NRAS、KRAS和CBL等骨髓增生异常综合征、JMML相关基因突变，外周血单核细胞百分比及绝对计数正常，后续随访过程中需注意JMML、AML等血液系统恶性肿瘤的可能。此外，患者父亲42岁，与患者基因型相同，但无血细胞减少（其父亲血常规示WBC 8.09×10^9^/L，ANC 5.31×10^9^/L，HGB 138 g/L，PLT 209×10^9^/L）。综上所述，本例患者的造血衰竭难以用NS一元论解释，我们考虑患者同时存在获得性再生障碍性贫血。至此，该患者诊断为获得性再生障碍性贫血合并NS。

针对再生障碍性贫血，予以司坦唑醇每次2 mg每日3次；艾曲波帕75 mg/d；环孢素A 50 mg 每12 h 1次。治疗1个月，患者血细胞计数上升。至2021年3月末次随访，血常规示WBC 6.1×10^9^/L，ANC 4.6×10^9^/L，单核细胞比例6.2％，单核细胞绝对计数0.4×10^9^/L，HGB 126 g/L，PLT 133×10^9^/L。未再发生出血表现，恢复正常生活及学习。

## 总结

该少年男性因血细胞减少首诊于血液科，骨髓检查提示骨髓造血不良，因明确的隐睾病史及躯体发育异常，初步疑诊IBMF。但二代基因测序检测到PTPN11突变，为文献报道过的NS热点突变，变异来自其父亲，未发现IBMF相关基因突变。结合患者及其父亲临床表型，确诊NS。然而患者同时伴发的BMF无法用NS一元论解释，故而诊断NS伴发获得性再生障碍性贫血。经免疫抑制及干细胞刺激治疗，患者血小板计数上升且出血倾向改善，表明BMF治疗有效，支持这种考虑。

IBMF常伴发躯体畸形，因而伴有发育迟缓和躯体畸形的年轻成人和儿童BMF，必须首先考虑IBMF。然而，并非所有IBMF均伴有发育异常，伴有躯体畸形的BMF也并非均为IBMF。规范的筛查试验、引致躯体畸形或骨髓衰竭的相关基因识别，以及仔细的家族成员临床表现型评估，对于厘清BMF与躯体畸形的关系和明确诊断更为重要。

